# First genomic prediction and genome‐wide association for complex growth‐related traits in Rock Bream (*Oplegnathus fasciatus*)

**DOI:** 10.1111/eva.13218

**Published:** 2021-03-17

**Authors:** Jie Gong, Ji Zhao, Qiaozhen Ke, Bijun Li, Zhixiong Zhou, Jiaying Wang, Tao Zhou, Weiqiang Zheng, Peng Xu

**Affiliations:** ^1^ Fujian Key Laboratory of Genetics and Breeding of Marine Organisms College of Ocean and Earth Sciences Xiamen University Xiamen China; ^2^ State Key Laboratory of Large Yellow Croaker Breeding Ningde Fufa Fisheries Company Limited Ningde China

**Keywords:** genome‐wide association, genomic selection, growth trait, *Oplegnathus fasciatus*

## Abstract

Rock Bream (*Oplegnathus fasciatus*) is an important aquaculture species for offshore cage aquaculture and fish stocking of marine ranching in East Asia. Genomic selection has the potential to expedite genetic gain for the key target traits of a breeding program, but has not yet been evaluated in *Oplegnathus*. The purposes of the present study were to explore the performance of genomic selection to improve breeding value accuracy through real data analyses using six statistical models and to carry out genome‐wide association studies (GWAS) to dissect the genetic architecture of economically vital growth‐related traits (body weight, total length, and body depth) in the *O*.* fasciatus* population. After quality control, genotypes for 16,162 SNPs were acquired for 455 fish. Heritability was estimated to be moderate for the three traits (0.38 for BW, 0.33 for TL, and 0.24 for BD), and results of GWAS indicated that the underlying genetic architecture was polygenic. Six statistic models (GBLUP, BayesA, BayesB, BayesC, Bayesian Ridge‐Regression, and Bayesian LASSO) showed similar performance for the predictability of genomic estimated breeding value (GEBV). The low SNP density (around 1 K selected SNP based on GWAS) is sufficient for accurate prediction on the breeding value for the three growth‐related traits in the current studied population, which will provide a good compromise between genotyping costs and predictability in such standard breeding populations advanced. These consequences illustrate that the employment of genomic selection in *O*.* fasciatus* breeding could provide advantages for the selection of breeding candidates to facilitate complex economic growth traits.

## 1 INTRODUCTION

The rock bream *Oplegnathus fasciatus*, a subtropical and carnivorous teleost fish belonging to the family Oplegnathidae, is primarily a dweller of estuaries throughout China, Korean Peninsula, Japan, and Hawaii (Schembri et al., 2010). They are also called barred knifejaw or striped beakfish owing to the stripes on their body surface. *O*.* fasciatus* is not only a popular game fish in coastal rocky shores (Schembri et al., 2010; Xiao et al., 2019), but also a potential commercially valued species in terms of offshore cage aquaculture and fish stocking of marine ranching in East Asia (Xu et al., 2013). And the ex‐factory price of *O*.* fasciatus* has reached up to 30 dollars per kilogram in China Park et al., 2018). The meat of *O*.* fasciatus* is considered as a delicacy, eaten as sashimi, or dried fillet (Park et al., 2018). Moreover, it is rich in many essential amino acids, lecithin, and collagen for human beings, which is much more nutritious than other fish species and highly loved by consumers (Wang et al., 2003). However, some factors, such as overfishing and environmental changes, are affecting fish yield and cost, especially in wild conditions (Shin et al., 2018). To get over these hindrances and meet consumer needs, *O*.* fasciatus* is produced via artificial aquaculture to achieve sustainable and cost‐efficient production. So far, as a developing aquaculture, the *O*.* fasciatus* breeding program was carried out based on phenotypic (e.g., body size) selection every year, so it is hopeful to make encouraging progress in genetic improvement.

As one of the most important economic traits for breeding, growth is a complex physiological process controlled by genetic and environmental factors (Wu et al., 2019). Owing to rock bream inbreeding in the rapid development of aquaculture industry, the genetic diversity decline, and genetic resource degradation, thus the growth of *O*.* fasciatus* is susceptible to virus infection (e.g., iridovirus) (Li et al., 2011; Zhang et al., 2014). Thence, there is an urgent demand to improve the genetic potentials of the cultured stocks. By making use of genomic tools to improve accuracy of estimated breeding values (EBV) and potentially identify causes that affect key production traits, the selective breeding can be notably elevated compared with classical pedigree‐based selection (Hickey et al., 2017; Meuwissen et al., 2013). Genomic selection (GS) demands no significant test, thus avoids biases in marker effect estimates and could accelerate the breeding cycle (Hill, 2013). In addition, GS using dense SNP markers will be the most suitable means to incorporate the genomic information to largely promote the genetic breeding progress for target traits and reduce the costs of breeding projects (Yoshida et al., 2018). To date, GS method has been widely applied to in livestock and plants (Campos et al., 2015; Cros et al., 2015; Heslot et al., 2015; Legarra et al., 2011; Longin et al., 2015; Meuwissen et al., 2016). In aquaculture species, the GS method beyond the classical pedigree‐based selection was adopted for growth traits and disease resistance, such as Atlantic salmon (Tsai et al., 2015), large yellow croaker (Zhao et al., 2020), gilthead sea bream (Palaiokostas et al., 2016), rainbow trout (Vallejo et al., 2017), and Japanese flounder (Liu et al., 2018). Although the promising commercial future of rock bream to global aquaculture, few studies have yet appraised the potential of genomic selection for breeding value prediction in this species.

The prediction performance is fundamental for successfully adopting GS methods. Different GS methods have been examined, and the prediction accuracy varies depending on which method was used, which mainly differ in relation to the assumption about marker effects and genetic relationship matrix calculation. Until now, there are numerous available GS methodologies. And the methods used most widely are GS models based on dense genome‐wide SNP markers, including genomic best linear unbiased predictor (GBLUP) and Bayesian methods. The GBLUP assumes that all marker effects come from a normal distribution and the genetic relationship matrix is merely calculated using genomic information (Vanraden, 2008). In contrast, Bayesian methods presume more flexible and non‐normal distribution of marker effects. For example, Bayes A (Meuwissen et al., 2001) model assumes that marker effects have heterogeneous variances. Bayes B (Meuwissen et al., 2001) model considers that a proportion of markers have non‐null effects, which is in contrast to Bayes A, because the approach includes the selection of covariates (SNP markers) that do not contribute to genetic variance. In Bayes C (Habier et al., 2011) approach, it is assumed that the marker effects have a common variance. And it includes marker selection with parameter π, which is defined as the probability of a SNP marker having a null effect. The Bayesian Lasso (BayesL) (Park & Casella, 2008) method adopts indirectly marker selection, since the marginal distribution of the markers follows a double exponential distribution, providing strong shrinkage of the marker effects to close to zero for large number of markers. Bayesian Ridge‐Regression (BayesRR) (Park & Casella, 2008) is the Bayesian version of GBLUP and assumes that all marker effects have the same variance component. In general, Bayesian methods usually surpass GBLUP method for the traits which are affected by a few large QTL. For traits that are affected by multiple mini‐effect QTL, GBLUP would likely perform better than or similar to the Bayesian methods (Chen et al., 2014). Therefore, by comparing the accuracy of different GS methodologies, we can determine which method can achieve higher accuracy for the genetic evaluation of economic growth traits in rock bream.

Despite great commercial value in this maricultured fish species, the molecular genetic mechanism underlying the growth of rock bream has not yet been fully understood. Previous genetic linkage analysis for the growth traits of aquatic animals was conducted by amplified fragment length polymorphism (AFLP), simple sequence repeat (SSR), microsatellite markers, and quantitative trait loci (QTL) analysis (Jongoh et al., 2014; Sun et al., 2013; Yu et al., 2016; Yue, 2014). However, these studies are restricted by sparse polymorphic molecular marker mapping. Double‐digest restriction site‐associated DNA (ddRAD) based on the high‐throughput next‐generation sequencing reduces DNA complexity by digesting genomic DNA with two specific restriction enzymes simultaneously (Peterson et al., 2012). This approach concurrently detects and genotypes SNP in multiple samples without a high‐density SNP genotyping array or a reference genome, which could obtain a considerable amount of genome‐wide molecule marker data (Gonzalezpena et al., 2016). As a consequence, genome‐wide association studies (GWAS) are employed to assess the association between SNPs dispersed throughout the genome and complex traits of interest. GWAS has cost‐effective and high‐resolution features, which may be able to resolve some shortfall of previous molecular genetic markers, such as the probable omission of QTL on account of inadequate marker density (Tsai et al., 2015). It is hard and costly to map functional genes in genomic level because of the polygenic nature of growth‐related traits. Nevertheless, the combination of GWAS and ddRAD sequencing could be a cost‐effective and convenient approach for accurate localization and identification of growth‐related traits in fish. Additionally, GWAS strategy for growth traits has been applied in various aquaculture fish species, including Atlantic salmon (Gutierrez et al., 2015; Tsai et al., 2015; Yoshida et al., 2017), rainbow trout (Gonzalezpena et al., 2016), common carp (Chen et al., 2018), catfish (Geng et al., 2017), and large yellow croaker (Zhou et al., 2019).

As far as we know, no research on performing GS and GWAS in a rock bream breeding population has yet been published. It is significant to detect QTL associated with growth trait and further assess the potential of genomic selection for growth traits in rock bream. In this study, we firstly report the results of conducting GS and GWAS using the data of 455 breeding population for evaluating prospects on GS implicating growth‐related traits and uncovering the inherited molecular mechanism in rock bream. First of all, heritability and genomic predict ability for growth traits were estimated using six different approaches (GBLUB and five Bayesian methods) to assess the potential of genomic selection for genetic improvement. Furthermore, the impact of variable numbers of SNPs and different SNP sampling schemes based on the position of the genome on genomic prediction were also evaluated for improving the genotyping cost‐efficiency. Finally, a genome‐wide association analysis was carried out on the same datasets to detect individual SNP or chromosome associated with growth traits and identify putative growth‐related genes.

## 2 MATERIALS AND METHODS

### 2.1 Experimental fish and sample collection

The *O*.* fasciatus* population in our study were from a commercial breeding company, Ningde Fufa Aquatic Breeding (Fujian, China). The population came from year‐class 2018, April spawning population. They were offspring of 260 randomly selected broodstocks with a random mating design. The offspring were firstly reared in a 40‐m^2^ (2.4 m in depth) cement pool and were fed chlorella and rotifers twice a day according to hatchery operation at birth, with aeration at 21 ~ 24°C in flow‐through seawater (27–33‰, DO ≥ 5 mg/L, pH = 7.6−8.4 and [NH_4_^+^ ‐N] ≤ 0.3 mg/L) under natural photoperiod conditions. After 8 months, the progenies were transferred to the marine cage and treated following guidance in standard culture protocols. At the age of 13 months, a total of 500 individuals were randomly collected and the growth‐related traits including body weight (BW), total length (TL), and body depth (BD) were measured. The dorsal fin of each fish was then collected and stored in anhydrous ethanol for DNA extraction.

### 2.2 DNA extraction and ddRAD libraries construction

Genomic DNA was extracted from dorsal fin using standard phenol–chloroform protocol (Sambrook & Russell, 2006) and quantified by Qubit 4.0 Fluorometer (Invitrogen). After integrity examination with 1.2% agarose gel electrophoresis, a total of 480 DNA samples met the quality requirement for ddRAD library construction. Ten ddRAD libraries were constructed by multiplexing 480 individuals following the protocols described previously (Peterson et al., 2012). Briefly, about 1.5–2 μg of genome DNA from each fish was digested with *EcoRI* and *MspI* (New England Biolabs; NEB). The P1 adapter with forward amplification primer and a 5 bp barcode was added to the *EcoRI* overhang, and the P2 adapter with reverse amplification primer was added to the *MspI* overhang, respectively. The DNA fragments of 300–400 bp were retrieved on E‐Gel (Thermo Fisher Scientific) and then amplified with 20 cycles of PCR with regular forward primer and indexes ligated reverse primers, followed by purifying with AMPure XP Beads (Beckman Coulter). The obtained ddRAD libraries were sequenced on an Illumina Hiseq2000 platform with 150‐ bp pair‐end strategy at Novogene Corporation.

### 2.3 SNP identification and quality control

The sequencing clean reads in every library were assigned to different individuals based on the barcode and index using the STACKS software (Catchen et al., 2013). Then, the clean reads of each individuals were aligned to the *O*.* fasciatus* genome (BioProject: PRJNA486885) by using BWA program (Li & Richard, 2010). After that, the program Populations in STACKS was used to carry out genotyping work. The filtering criteria for SNPs using PLINK v1.90 (Purcell et al., 2007) were set as individuals calling rate >90%, SNP loci calling rate >90%, and minor allele frequency (MAF) <5%. Missing genotypes were inputted with the software BEAGLE4 (Browning & Browning, 2007). Haploview software was used to find tagging SNP because there may be a high correlation among the adjacent SNP loci, which would reduce the multicollinearity of the GS model (Barrett et al., 2005). As previous chromosome‐level genome study of *O*.* fasciatus* (GigaDB database: PRJNA486885 and PRJNA486572) (Xiao et al., 2019) only released the contigs, we selected the syntenies large yellow croaker genome (BioProject: PRJNA505758) as a reference to construct *O*.* fasciatus* pseudo‐chromosomes. The contigs of *O*.* fasciatus* were mapped and assembled onto the chromosomes based upon the large yellow croaker genome to facilitate further genetic analysis.

### 2.4 Genome‐wide association study and candidate gene annotation

The GWAS were performed for the three growth‐related traits using two approaches. Firstly, population structure analysis was performed before GWAS using PLINK (‐‐cluster ‐‐matrix). Principal component analysis algorithm could estimate the potential genetic relatedness and display population structure of the 455 samples using 16,162 SNPs (~16 K SNP panel). Population structure identified by PCA with the first three principal component factors (Figure [Link], [Link], [Link], [Link], [Link]). Accommodating for population stratification and relatedness, univariate liner mixed model (ULM) by GEMMA, was used for association studies. Meanwhile, multiple locus mixed linear model (MLMM) was also used for GWAS by GAPIT (Lipka et al., 2012). Wald frequentist test was chosen to test for significance, and Bonferroni threshold for *p* < 0.05 was determined as genome‐wide significance. The Manhattan plot of the −log_10_ (*p*‐value) and QQ‐plot were produced in R software.

Considering growth trait is a quantitative trait affected by multiple genetic loci, the Bonferroni test (0.05/number of QC‐filtered SNPs) criterion is extremely strict to be a threshold. In this study, given that none of the SNPs from GEMMA and GAPIT reached the Bonferroni threshold for *p* < 0.05, the threshold of suggestive association was arbitrarily set to −log_10_ (*p*) > 4.0 because the Bonferroni test (0.05/numbers of SNPs) criterion was extremely strict to be a threshold, considering GWAS was hypothesis generating (Wang et al., 2012; Yang et al., 2011). To annotate the candidate genes, the ±100 K bp genome regions adjacent to the significant associated SNPs were scanned and the candidate genes were annotated accurately by BLAST against the non‐redundant protein database.

### 2.5 Genetic parameter estimation

Analysis of each trait was performed using the R package sommer (Covarrubias‐Pazaran, 2016) and BGLR (Perez & Campos, 2014) with the following mixed linear model:

(1) y=Xb+Zu+e

where y is the vector of observed phenotypes for different traits; b vector is the fixed effects; X and Z represent the corresponding incidence matrices for fixed effects and genetic effects, respectively; U is the vector of random additive effects with the following distribution $N\;(0,\;G\sigma_{g}^{2})$, where G is genomic relationship matrix (Vanraden, 2008) for certain analyses as described below and $\sigma_{g}^{2}$ is additive genetic variance. And e is the vector for residual error with the distribution $N\;(0,I\sigma_{e}^{2}$ **)**, where I is a vector of identity matrix and $\sigma_{e}^{2}$ is the residual variance. Hence, the narrow sense heritability $\left(h^{2}\right)$ is computed by the additive genetic variance and total phenotypic variance via the following formula:

(2) $$h^{2}=\frac{\sigma_{g}^{2}}{\sigma_{g}^{2}+\sigma_{e}^{2}}$$

The model with the same fixed and random effects is as in Equation (1). The $h^{2}$ is the heritability, and the sum of $\sigma_{g}^{2}+\sigma_{e}^{2}$ is phenotypic variance. All model parameters and marker effects estimated in a Bayesian framework were estimated using the Gibbs sampling algorithm implemented in BGLR package. The mixture parameter π was set to 0.95 in the GS analysis with BayesB and BayesC. A total of 50,000 iterations were implemented with a burn‐in period of 5000 cycles. And the thinning interval was set as five iterations.

### 2.6 Cross‐validation for different model comparisons

Predictive abilities of the different models described above (GBLUP, BayesA, BayesB, BayesC, BayesL, BayesRR) were assessed through a fivefold cross‐validation scheme. The 455 individuals were randomly and evenly split into sequential non‐overlapping training population (*n* = 364) and testing population (*n* = 91) with the ratio of 4:1. The training population was used to build the model, and the phenotype record in the testing population was masked and used to validate the effectiveness of established model. In each replicate, the same combination of training population and testing population was adopted to perform the model prediction, so the results would have sufficient comparability for the all algorithms. The fivefold cross‐validation procedure was repeated 40 times in order to reduce randomly sampling effects, and average values of predictability were calculated. And we evaluated the predictability for the six algorithms based on the full data (~16 K SNP panel), where the predictability was calculated as the correlation coefficient between GEBVs and phenotypes.

### 2.7 Predictability at varying marker density

The GBLUP model was fitted using different subsets of SNPs of various sizes. We selected three different criteria for evaluating the potential of different marker densities for genomic prediction as described below. The first method (i) was based on the informative SNPs that were selected according to *p*‐values in ascending order by GWAS which implemented via Gemma software. Secondly, (ii) the same numbers of SNPs were randomly selected from the ~16 K SNP panel. Finally, (iii) the reduced‐density SNP panels were developed by sampling evenly spaced SNPs from the 16 K SNP panel trying to optimize the genome coverage across the chromosomes. All three scenarios adopted a cumulative approach. For instance, beginning with the smallest subset (50) of SNPs, additional 50 SNPs were added to the previous set each time until reached the biggest (total) subset. For each SNP subset, the GS model was built and corresponding predictability was calculated through fivefold cross‐validation.

## 3 RESULT

### 3.1 Summary statistics and heritability estimation

In this study, a total of 10 ddRAD‐seq libraries were constructed in 480 samples of *O*.* fasciatus*. In the procedure of genotyping, three statistical parameters were estimated and listed as follows: Average sequencing depth was 35.3×, average reference genome mapping ratio was 91.6%, and average genome coverage was 1.7%. After filtering both individuals and SNPs with low quality, missing genotype imputation, and searching for tagging SNP, the eventual dataset utilized for the GS and GWAS analysis composed of 16,162 QC‐filtered SNPs genotyped in 455 samples. The growth‐related phenotype (BW, TL, and BD) was measured at approximately 1 year post‐hatching. The SNP density on each chromosome was shown in Figure [Link], [Link], [Link], [Link], [Link]. The mean and standard deviation values for the growth‐related traits were 130.64 ± 32.43 g, 18.11 ± 1.4 cm, and 7.08 ± 0.58 cm for BW, TL, and BD, respectively. The strong and extremely significantly positive correlations among these three growth traits reflected the predictability of trait measurement. The genetic correlation between the three traits was higher than 75% for all traits, which is slightly lower than the phenotypic correlation (~80%) (Table 1). Estimates of genomic heritability varied depending on the method used, with GBLUP yielding the closely heritability values as those obtained using Bayesian methods for BW traits. For TL and BD, however, the genomic heritability in BayesL method was considerably lower than the other methods. When using BayesRR, the heritability of BD was highest (0.28). The variance components (*V*_g_ and *V*_e_) had different estimates with distinct methods used (Table 2). Combining the results across all genomic methods, the average heritability for *O*.* fasciatus* (~0.38 for BW; ~0.33 for TL; ~0.24 for BH) was at moderate level for all traits. Interestingly, the heritability of GBLUP in all traits had the most similar estimates with BayesB methods used.

**TABLE 1:** Statistical results for phenotypic and genetic parameter for the growth‐related traits in *O*.* fasciatus*

	BW	TL	BD
Mean (SD)	130.71 ± 32.88 g	18.12 ± 1.41 cm	7.07 ± 0.59 cm
Correlation			
BW	1	0.88	0.85
TL	0.88	1	0.81
BD	0.79	0.76	1

Note

Genetic correlation was estimated based on the genomic relationship matrix, and values are shown below the diagonal, while phenotypic correlation values are shown above the diagonal.

**TABLE 2:** Estimates of variance components for three growth traits of *O*.* fasciatus* using different GS models

	BW	TL	BD
GBLUP			
h^2^ (SD)	0.39 (0.09)	0.36 (0.09)	0.23 (0.08)
V_g_ (SD)	454.89 (126.50)	0.77 (0.23)	0.08 (0.03)
V_e_ (SD)	709.52 (83.19)	1.37 (0.16)	0.28 (0.03)
BayesL			
h^2^ (SD)	0.42 (0.02)	0.25 (0.06)	0.19 (0.05)
V_g_ (SD)	457.23 (37.52)	0.50 (0.13)	0.06 (0.02)
V_e_ (SD)	641.58 (33.63)	1.48 (0.12)	0.28 (0.02)
BayesRR			
h^2^ (SD)	0.36 (0.07)	0.36 (0.07)	0.28 (0.05)
V_g_ (SD)	394.56 (78.26)	0.72 (0.15)	0.10 (0.02)
V_e_ (SD)	694.77 (69.59)	1.29 (0.14)	0.25 (0.02)
BayesA			
h^2^ (SD)	0.38 (0.08)	0.34 (0.07)	0.24 (0.06)
V_g_ (SD)	416.69 (96.51)	0.67 (0.15)	0.09 (0.02)
V_e_ (SD)	678.36 (81.14)	1.33 (0.14)	0.27 (0.02)
BayesB			
h^2^ (SD)	0.34 (0.05)	0.34 (0.09)	0.25 (0.06)
V_g_ (SD)	372.18 (60.00)	0.69 (0.19)	0.09 (0.02)
V_e_ (SD)	716.08 (57.33)	1.32 (0.16)	0.26 (0.02)
BayesC			
h^2^ (SD)	0.36 (0.07)	0.34 (0.07)	0.26 (0.06)
V_g_ (SD)	393.84 (83.69)	0.68 (0.15)	0.09 (0.02)
V_e_ (SD)	696.95 (72.84)	1.32 (0.14)	0.26 (0.02)

Note

Standard errors in brackets.

Abbreviations: h^2^, estimated heritability; V_g_, additive genetic variance; V_e_, residual variance.

### 3.2 Genome‐wide association studies and putative gene identification

**FIGURE 1:**
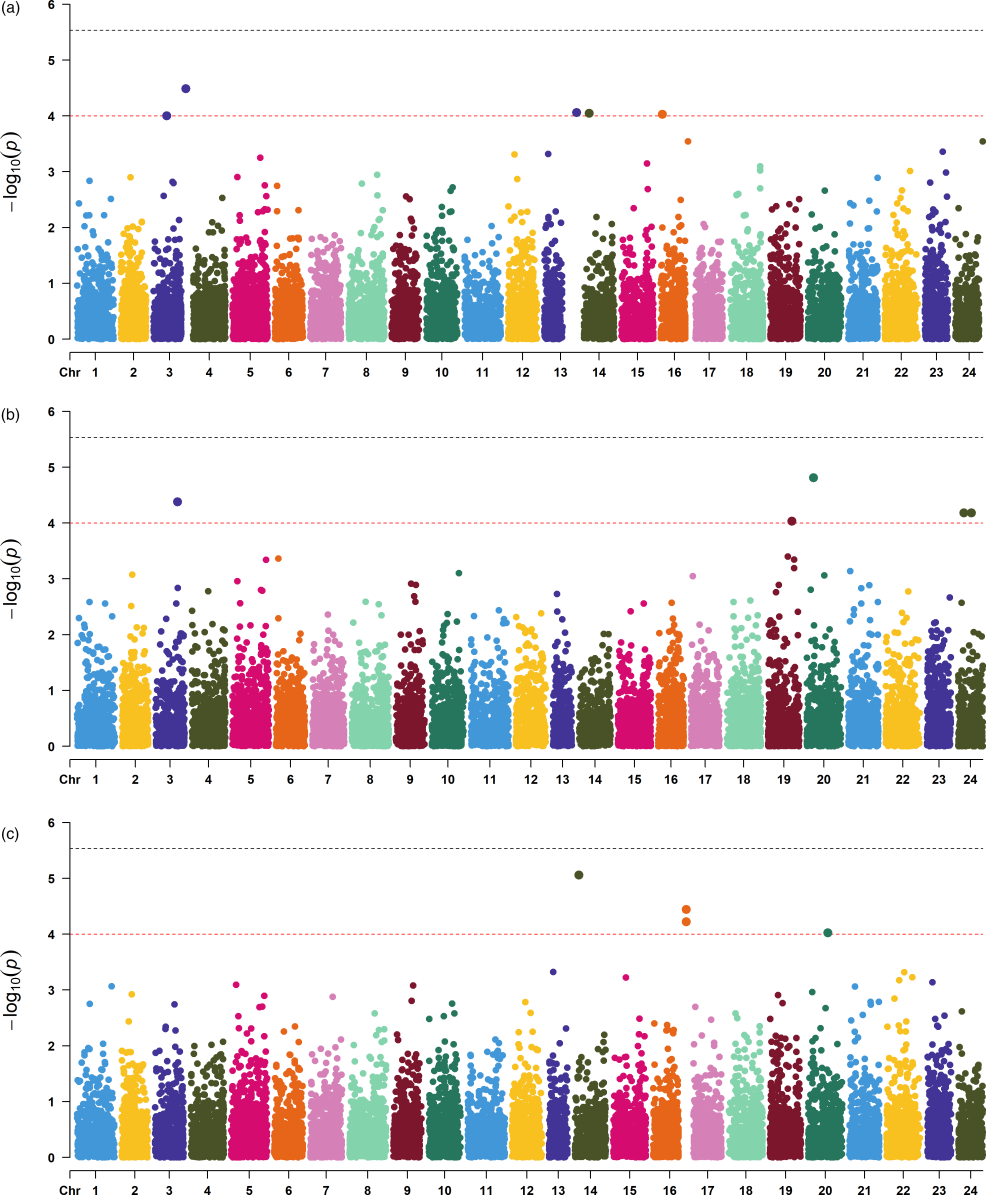
Manhattan plot for the significance of each genetic variant in GWAS for (a) body weight (BW), (b) total length (TL), and (c) body depth (BD). The black line denotes the genome‐wide significance threshold, and the red line denotes the suggestive threshold

A normal or near‐normal distribution was observed in the association population for the three investigated traits (Figure [Link], [Link], [Link], [Link], [Link]). There was no significant association detected by Gemma or GAPIT between any SNP and any of the three analyzed BW, TL, BD traits. Manhattan plots and QQ plots were shown in Figure 1 and Figure S4, respectively. Fourteen of the 16,162 SNPs surpassed a suggestive threshold (relaxed threshold) and were used for determining putative candidate genes in BW, TL, and BD, respectively. Five SNPs located on Chr3, Chr13, Chr14, and Chr16 were identified to be associated with BW, and the most significant one was SNP12834 on Chr3 (*p* = 3.26 × 10^−5^; Table S1). The *p*‐value, allele frequency, and proportion of genetic variation explained (PVE) for the top markers in three traits were also given in Table S1. The PVE by the top markers of three straits ranged between 5.69% and 7.55%. To identify genes potentially associate with growth traits, we screened *O*.* fasciatus* reference genome regions based on the above 14 SNPs. From these 14 SNPs, six, thirteen, and eleven genes were annotated corresponding to BW, TL, and BD, respectively (Table S2). Some candidate genes exceeding suggestive threshold have been suggested to be involved with growth‐related traits in previous studies. For BW, both nutritionally regulated adipose and cardiac‐enriched protein (*NRAC*) and kinesin‐like protein KIF26A (*KIF26A*) were identified on Chr3. Besides, E3 ubiquitin‐protein ligase HACE1 (*HACE1*) was identified on Chr14, which involved in early embryonic development (Iimura et al., 2016). For TL, we identified the genes nutritionally regulated adipose and cardiac‐enriched protein homolog (*NRAC*), unique cartilage matrix‐associated protein (*UCMA*), and regulator of G‐protein signaling 12 (*RGS12*) located in chromosome 3, 20, and 24, respectively (Table S2).

### 3.3 Predictability of GBLUP and Bayesian models

We compared and presented the evaluation of the predictive performance of GBLUP and five Bayesian methods using whole SNPs with a fivefold cross‐validation. In line with expectations, the predictive abilities followed the same tendency as the estimated genomic heritability. The predictive abilities estimated using GBLUP generated no obvious difference for particular traits to those obtained using different Bayesian methods (Table 3). For this rock bream population, both GBLUP and Bayesian genomic predictive abilities were medium, BW (averaging 0.30) exhibited the highest predictability across all traits, followed by TL (averaging 0.27), and BD (averaging 0.23) (Table 3), implying the existence of a larger quantity of additive genetic variation for these growth traits in this breeding population. The predictability among the six methods varied from 0.24 to 0.29 for TL, while the values for BW were from 0.29 to 0.31. Standard deviations of predictability range from 0.07 to 0.10 across traits and methods, where the highly predictable traits were not with smaller standard deviations than those low predictable traits. Among the six methods, GBLUP and BayesRR relatively outperformed the other methods which would be the more efficient methods in this dataset.

**TABLE 3:** Estimated predictability by ‘fivefold cross‐validation for three growth traits *O*.* fasciatus* using different GS models

	GBLUP	BayesL	BayesRR	BayesA	BayesB	BayesC
BW	0.31 (0.08)	0.29 (0.08)	0.31 (0.08)	0.30 (0.07)	0.31 (0.08)	0.31 (0.09)
TL	0.27 (0.07)	0.26 (0.10)	0.29 (0.07)	0.25 (0.09)	0.29 (0.08)	0.24 (0.08)
BD	0.25 (0.08)	0.20 (0.07)	0.24 (0.09)	0.23 (0.09)	0.23 (0.08)	0.25 (0.10)

Note

Standard errors in brackets.

### 3.4 Impact of SNP numbers on predictability

**FIGURE 2:**
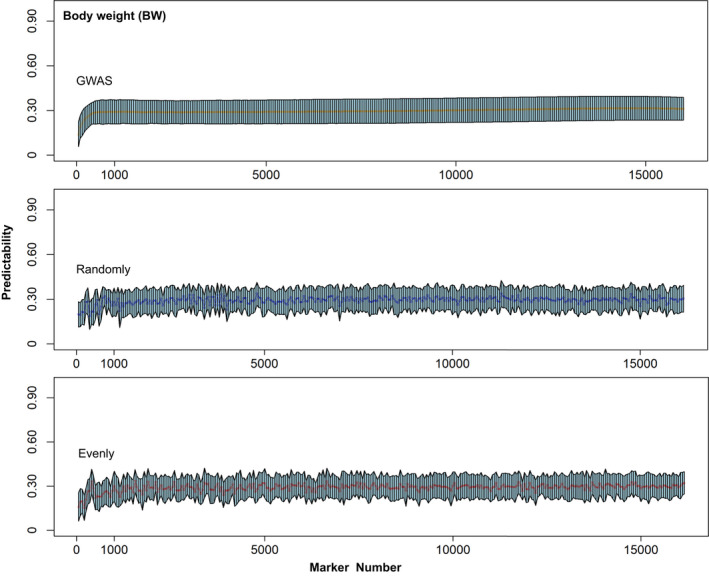
Estimates of predictability with increasing numbers of SNP for body weight (BW) using three cumulative approach to SNP sampling. The orange line indicated gradually increased markers that sorted by GWAS (*p*‐value in ascending order). The blue line indicated gradually increased markers that were randomly selected from all available SNPs. The red line indicated gradually increased markers that spaced evenly across the genome. The solid line indicated the mean value at each marker number, and the shaded area was formed by connected the dot of positive and negative standard deviations

**FIGURE 3:**
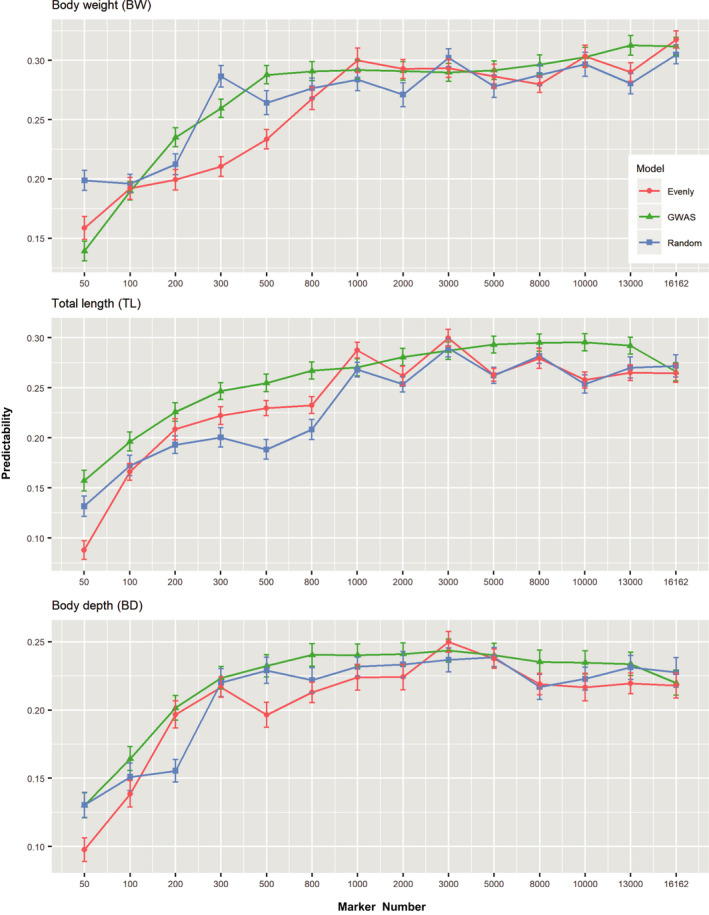
Genomic estimates of predictability for *O*.* fasciatus* using different SNP sampling methods. 14 SNP subsets were selected using GWAS informative, randomly and evenly sampled SNPs (0.05 K, 0.1 K, 0.2 K, 0.3 K, 0.5 K, 0.8 K, 1 K, 2 K, 3 K, 5 K, 8 K, 10 K, 13 K, ~16 K SNPs)

Based on results of the different prediction methods, only GBLUP was chosen to evaluate the influence of different density SNP schemes on the predictive ability. Estimates of predictive ability increased rapidly with increasing numbers of SNP up to ~800 for all traits (Figures 2 and 3; Figure [Link], [Link], [Link], [Link], [Link]). Predictive ability plateaued with approximately 1000 SNPs, although predictive abilities still increased slowly after that. Additionally, when less than 1000 SNPs were used, a much larger fluctuation in predictive ability was seen, especially in random and even sampling strategy. These results indicated that at least in this population, models with ~1000 SNPs will provide predictive ability equivalent to that by using all the available SNPs. There was no difference observed in the estimates of predictive abilities when different position‐based SNP sampling schemes (randomly and evenly methods) were used as long as the total number of SNPs was close to 1000. There was visible difference in predictability for any trait between model with SNPs sorted by GWAS and model with SNPs selected randomly or evenly methods. The predictive abilities estimated with a subset of evenly spaced SNPs were similar unstable tendency with those using randomly sampled SNPs in all traits (Figure 1; Figure S5). By contrast, the predictive abilities using informative SNPs sorted by GWAS showed a steady upward trend as the SNPs increasing. The result was observed for BW, where estimates of predictive ability by GWAS were less than randomly and evenly strategy when using little SNPs (50 SNPs) (Figure 3). In TL, the opposite result appeared in which GWAS method performed better than randomly and evenly tactics. However, when the marker density reaches 100 SNPs or higher, estimates of predictive ability using GWAS were gradually higher than randomly and evenly methods in all three traits (Figure 3).

## 4 DISCUSSION

### 4.1 Moderate heritability values for growth traits in rock bream

In selective breeding projects for *O*.* fasciatus*, body weight, total length, and body depth are considered to be important selection indexes for growth traits. Genomic heritability reflects the real genetic relationships among individuals; therefore, it corresponds to the proportion of phenotypic variance that can be explained by regression on molecular markers. Generally, GBLUP is the most robust method and supplies higher predictability for highly polygenic traits, while the Bayesian methods are more applicable to traits determined by major genes (Wang et al., 2018). Judging from the result, moderate heritability values for BW, TL, and BD in rock bream were identified, which were estimated using genomic information, for the first time in *Oplegnathus*. From the comparison of different prediction methods, we found that Bayesian methods (except BayesL) performed equally well as GBLUP method for the real dataset of *O*.* fasciatus*. These five methods had good stability and were qualified to predict the heritability of the population accurately. These estimates are the first heritability estimates of weight/length for rock bream with genomics matrices.

### 4.2 Candidated loci and genes for growth traits

In the current study, no evidence of major quantitative trait loci was found for the three analyzed traits in rock bream. There were no SNPs surpassed the stringent genome‐wide significance threshold of all traits. Our results are consistent with previous studies in which very few obviously associations had shown for growth‐related straits by GWAS. For example, polygenic regulation of growth traits in the giant grouper was estimated that only 1.38% and 0.95% of genetic variance were explained by the main 20 SNP windows for body weight at 10 and 13 months (Gonzalezpena et al., 2016). Tsai et al. (2015) found an accumulated genetic variance of 0.63% explained by the top 10 SNPs located in different chromosomes for body weight of Atlantic salmon in a GWAS using a dense SNP array. Geng et al. (2017) utilized GWAS for identification of QTLs for body conformation in catfish, in which no significant QTLs were identified for body depth and body breadth, but three suggestive QTL regions were identified for BD on LG 5, LG 13, and LG 14. Therefore, we assumed that early growth traits in rock bream were a polygenic architecture in nature. Additionally, the proportion of variance explained (PVE) by top SNP was relatively low (up to ∼7%), highlighting the absence of any major QTL controlling juvenile growth traits in this population. These very stringent tests typically result in only the largest effect QTLs being found, while the great majority have too small effects to be detectable in the limited power GWAS populations used. If no major effects exist, then no associations would be found, which is most likely the case of the limited association results for growth targeted in our study.

In order to deploy genetic markers in commercial aquaculture breeding programs, it is essential to first determine the genetic structure of the aim trait. A potential criticism to our GWAS is that population sizes and marker density used in the current rock bream population were relatively small, such that experiments have suffered from low power to detect the likely large number of small effect loci controlling growth‐related traits. Meanwhile, with a polygenic or oligogenic structure and SNPs explaining small proportion of the additive genetic variance, genomic selection for growth‐related traits in this rock bream population is more effective than marker‐assisted selection (MAS). Using MAS with such an architecture would not supply satisfactory consequence given that only a small proportion of variance can be explained by individual SNPs. In order to provide useful GWAS data toward breeding for growth traits in rock bream, it will be necessary first to massively increase the sample sizes and use a substantially higher density of SNPs, such that adequate power is facilitated to detect at least part of the slightly larger effects segregating in the aim breeding population.

Furthermore, from the top SNPs that explained the higher proportion of genetic variance, we identified several genes that could potentially be involved in growth trait. It is worthy to mention that some of the most biologically relevant candidates maybe provide reference for future studies in rock bream. For instance, *HACE1* plays an important role in early developmental processes in *Xenopus laevis*, which control body axis elongation through regulation of convergent extension (Iimura et al., 2016). *KIF26A* is a rather atypical member of kinesin superfamily, which plays a key role in enteric nervous system development by repressing a cell growth signaling pathway (Zhou et al., 2009). For both BW and TL, we identified gene *Nrac* that was localized to the plasma membrane, and highly induced during adipocyte differentiation with potential functions in metabolism (Zhang et al., 2012). In addition, *UCMA* was identified as a secreted protein present in fetal and juvenile growth plate cartilage and is highly conserved between human, mouse, rat, dog, and guinea pigs (Surmannschmitt et al., 2008). The RGS protein family member *RGS12* was related to skeletal muscles of developing mouse embryos, suggesting a potential role for this unique RGS family member in the skeletal muscle developmental process (Martinmccaffrey et al., 2005).

### 4.3 Predictability of different GS models

The results from the examination of GS in the rock bream population used in the present study were encouraging, with predictability obtained through ‘fivefold cross‐validation analyses using GBLUP and five Bayesian methods being relatively high. Predictive abilities of growth traits using six different methods attained slightly different results for all traits, in consistent with previous reports in marine fish (Liu et al., 2019). These results confirmed complex architecture of growth traits in *O*.* fasciatus*, which were controlled by a large number of small effect loci and fit adequately the infinitesimal model. The predictability estimates obtained for growth traits in *O*.* fasciatus* using GBLUP and Bayesian methods were approximately in line with those reported for Yesso scallop (Dou et al., 2016) and Yellow drum (Liu et al., 2019). In addition to the influence of different algorithms, predictabilities were closely related to the effective population size, the number of individuals used for model training, and the limited genetic diversity available in species and especially in introduced population (Müller et al., 2017). In general, from the applied breeding perspective, the genomic predictabilities were better than the predictive abilities based on phenotypic data in many marine species (Barría et al., 2018; Garcia et al., 2018; Palaiokostas et al., 2018; Tsai et al., 2015). On the whole, there appears to be increasing consensus that the heritability of aim trait could affect the accuracy of the genomic prediction, and traits with high heritability could be predicted more accurately than those with low heritability (Daetwyler et al., 2010; Pszczola et al., 2012; Resende et al., 2012). In this study, we found the prediction reliability of BW was significantly higher than the prediction reliability of TL and BD for all six models, which also imply that BW had higher heritability than TL and BD in *O*.* fasciatus*.

The verification of the results in the present study of rock bream would be a logical next step. To predict the phenotype of selection candidates, the GS model with the highest predictability will be used to calculate GEBV for each fish. Then, we can sort the GEBV of all candidates in descending order and chose the top 50 individuals as broodstocks. The selected individuals will be transferred to a new indoor cement pool to reproduce, and their offspring were considered as improved strain that theoretically had higher growth performance. The remaining selection candidates were placed together to reproduce, and their offspring were treated as control strain representing the average growth level of local population of rock bream. The improved strain and control strain will be firstly cultivated in indoor cement pool for one month and then transferred into the offshore marine cage. The daily culture management follows the standard regime established by breeding company. At the time of 1 year post‐hatch, the growth‐related traits on hundreds of both improved strain and control strain will be measured and compared.

The limitation of the present study lies in the fact that the current rock bream population lacked well‐documented pedigree information. There is no means to compare the performance of pedigree‐based estimate with that of genomic prediction. Although it cannot be said directly whether genomic selection is worth pursuing because the comparison to pedigree is not there, genomic heritability and predictability, regardless of the method used, were generally higher than the pedigree‐based estimates in multitudinous fishes (e.g., Nile tilapia (Yoshida et al., 2019), catfish (Garcia et al., 2018), Atlantic salmon (Tsai et al., 2015), sea bream (Palaiokostas et al., 2016), rainbow trout (Vallejo et al., 2018), European sea bass (Palaiokostas, Cariou et al., 2018), and common carp (Palaiokostas, Kocour et al., 2018)). Furthermore, it has been shown that genome selection increased genetic gain and reduced the rate of inbreeding in many species such as pig (Lillehammer et al., 2011), chicken (Wolc et al., 2011), common carp (Palaiokostas, Kocour et al., 2018), and Atlantic salmon (Tsai et al., 2016). Although the preliminary results of genomic prediction in breeding for this species are encouraging and promising, further experiments are still necessary to build family and record pedigree information. Without a doubt, the supplement of pedigree information will verify whether incorporation of genomic selection brings about significant improvement in selection accuracy and genetic gain compared with traditional family selection in aquaculture breeding.

### 4.4 Effects of marker density on predictability

Compared to livestock, in the breeding candidates of aquaculture species, it is not worthy to apply costly high‐density genotyping approaches. The use of lowly cost sparse genotyping to improve predictability in a breeding program is important for implementation of GS. Indeed, SNP density and distribution are crucial for the performance of statistical models. It has been reported that SNPs chosen randomly across the genome might affect the stability of genomic prediction (Spindel et al., 2015). An appropriate increase in marker density and a more even distribution of SNPs may improve the accuracy and stability of genomic prediction. Thus, we have adopted three strategies for testing, SNPs sorted by GWAS (*p*‐values in ascending order); SNPs were randomly selected from all available SNPs and spaced evenly SNPs across the genome, respectively. Prediction models using ~1000 SNPs provided predictive abilities almost equivalent to using all available SNPs for all traits, and obvious difference was observed using different sets of SNPs. The predictive abilities using informative SNPs sorted by GWAS show a more stably upward trend compared with the predictability estimated by evenly spaced SNPs or randomly sampled SNPs on genome as the SNP numbers increasing. Reasons for this major divergence are comprehensible due to informative SNPs by GWAS which *p*‐value was sorted in ascending order. However, the other two strategies did not essentially follow the principle of *p*‐value sorted.

As shown in Figure 3, the predictive abilities were gradually higher with an informative SNP set than with a random SNP set or spaced evenly SNP set when SNP numbers achieved more than 100 SNPs, which suggested that informative SNPs by GWAS sorted had advance in genomic prediction. Moreover, random SNP set and spaced evenly SNP set seemly requires more SNPs to achieve similar predictive abilities to those achieved by using whole‐genome SNPs because of their poor stability. By contrast, GWAS informative SNPs could bring predictive ability close to those using whole‐genome SNPs. From previous studies in marine species, a small number of SNPs rather than whole‐genome SNPs for selection could be adopted to reduce cost of GS (Gutierrez et al., 2018; Tsai et al., 2015; Wang et al., 2017; Yoshida et al., 2019). Although these results indicate that both effects (the position and number of SNPs) are important to the accuracy of predictions, they also suggest that GWAS strategy might provide a big‐added advantage compared with random or evenly distribution sampling.

## 5 CONCLUSION

So far, this is the first study that contributes to experimental data supporting the positive prospects of genomic selection and GWAS to assess complex growth traits in *O*.* fasciatus*. The GWAS shows a polygenic architecture for BW, TL, and BD, with some markers explaining a small proportion of genetic variance, which indicates that the implementation of genomic selection is necessary for these traits in the present rock bream population. These three traits are found to be moderately heritable and display high genetic correlation. Comparison of six GS models reveals that GBLUP model subtly outperforms other Bayesian models in this breeding population. The predictive abilities using informative SNPs sorted via GWAS illustrate a more stably upward trend compared with the predictability estimated by evenly spaced SNPs or randomly sampled SNPs on genome as the SNP numbers increasing. The low SNP density (around 1 K selected SNP based on GWAS) is sufficient for accurate prediction on the breeding value for the three growth‐related traits in the current studied population, which will provide a good compromise between genotyping costs and predictability in such standard breeding populations advanced. The results from the present study demonstrate that GS has potential for substantial improvement in predictability, and ddRAD‐seq combined with GWAS seems to be an effective channel for examining and promoting a polygenic trait in the rock bream breeding population.

## CONFLICT OF INTEREST

The authors declare that they have no conflict of interest.

## Supporting information

Fig S1

Fig S2

Fig S3

Fig S4

Fig S5

Table S1

Table S2

## Data Availability

The SNP data have been uploaded to the NCBI Sequence Read Archive (SRA) under BioProject accessions PRJNA707324.
