# Supplementary figures and images for: First genomic prediction and genome‐wide association for complex growth‐related traits in Rock Bream (Oplegnathus fasciatus)
Source: Evol Appl. 2021 Mar 17;15(4):523–36. doi: 10.1111/eva.13218 (PMC9046763; doi:10.1111/eva.13218)

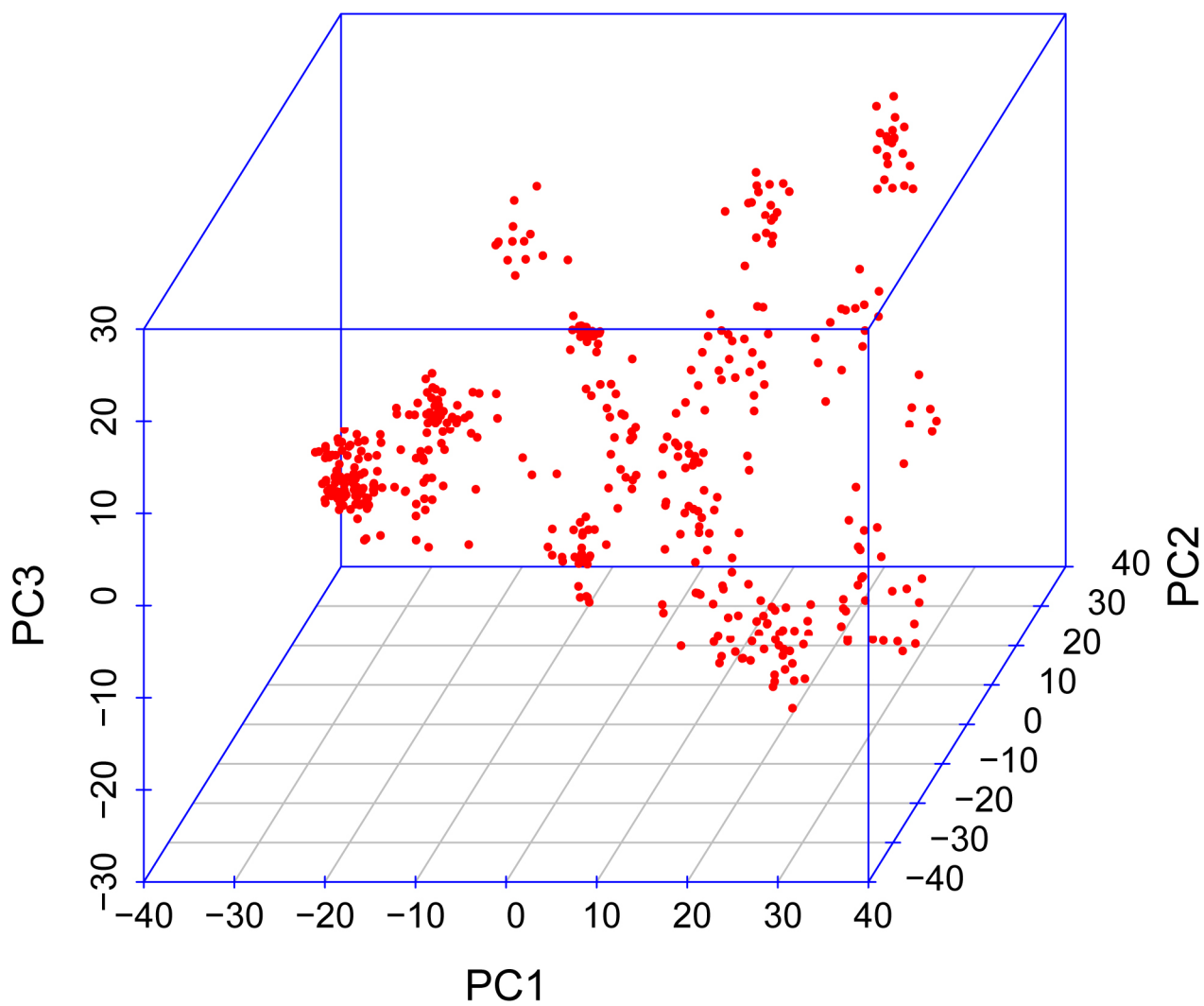

Supplement: Supplementary file 1 — Fig S1 [file EVA-15-523-s003.pdf]

# The number of SNPs within 1Mb window size

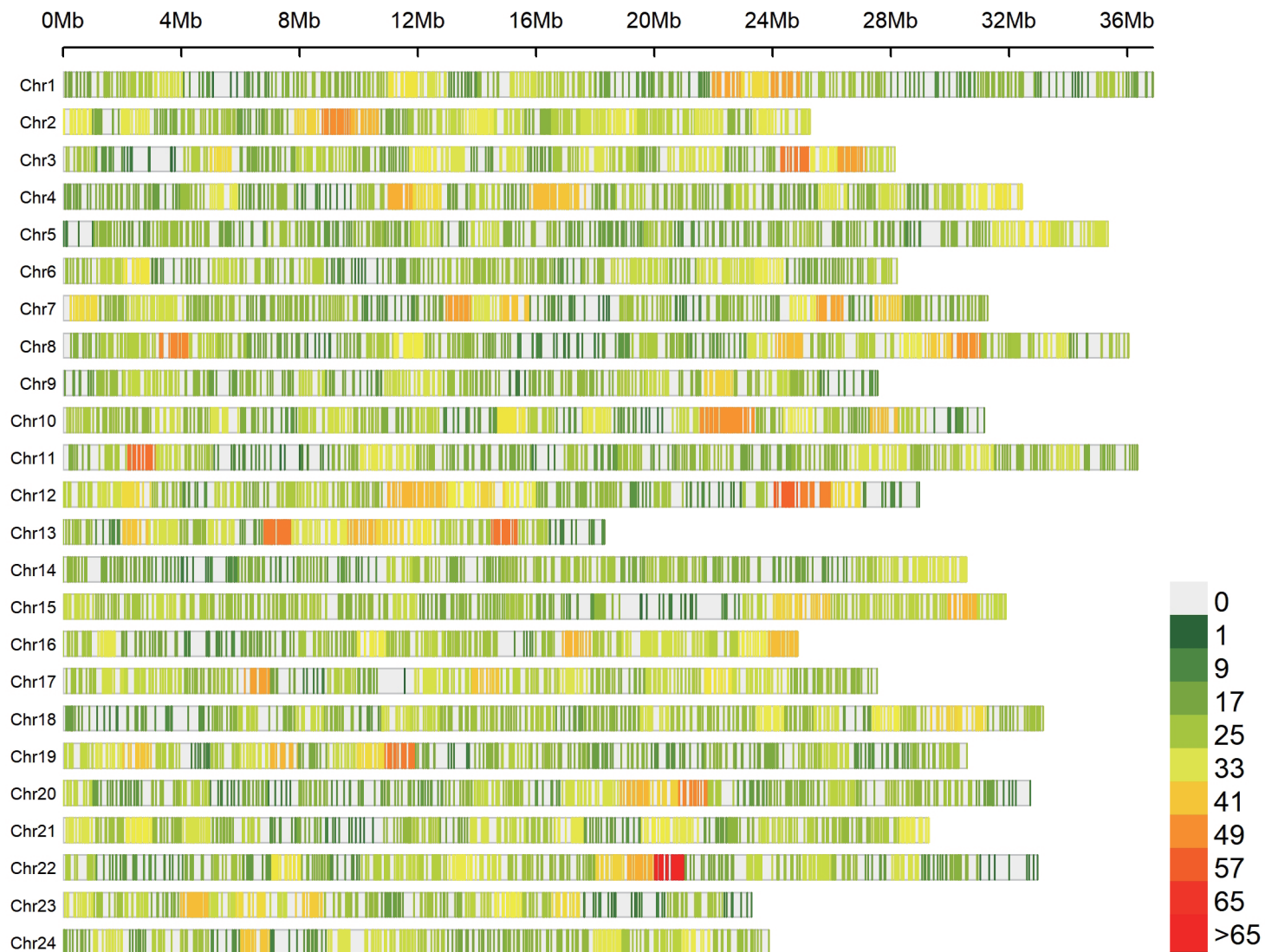

Supplement: Supplementary file 2 — Fig S2 [file EVA-15-523-s005.pdf]

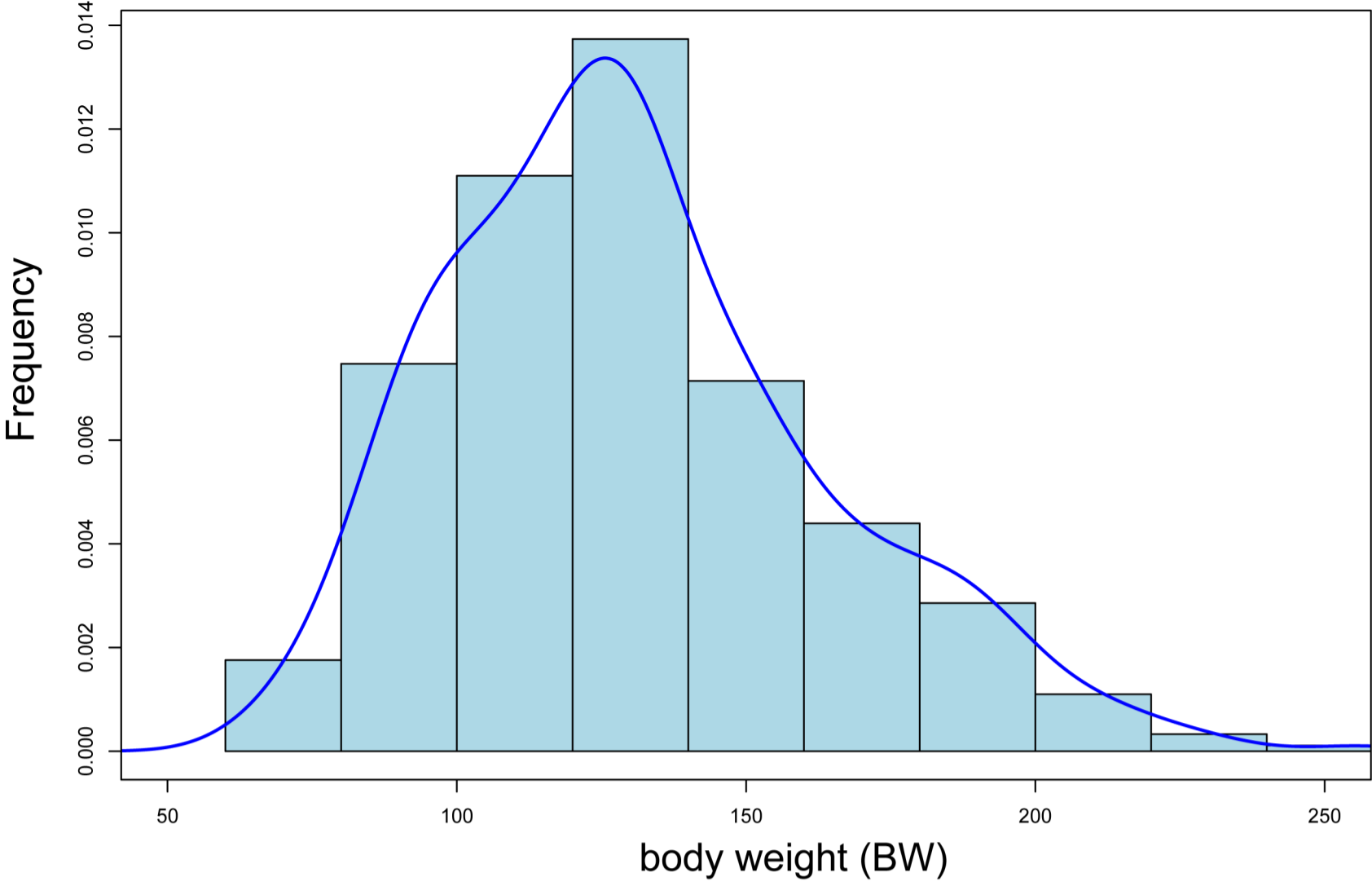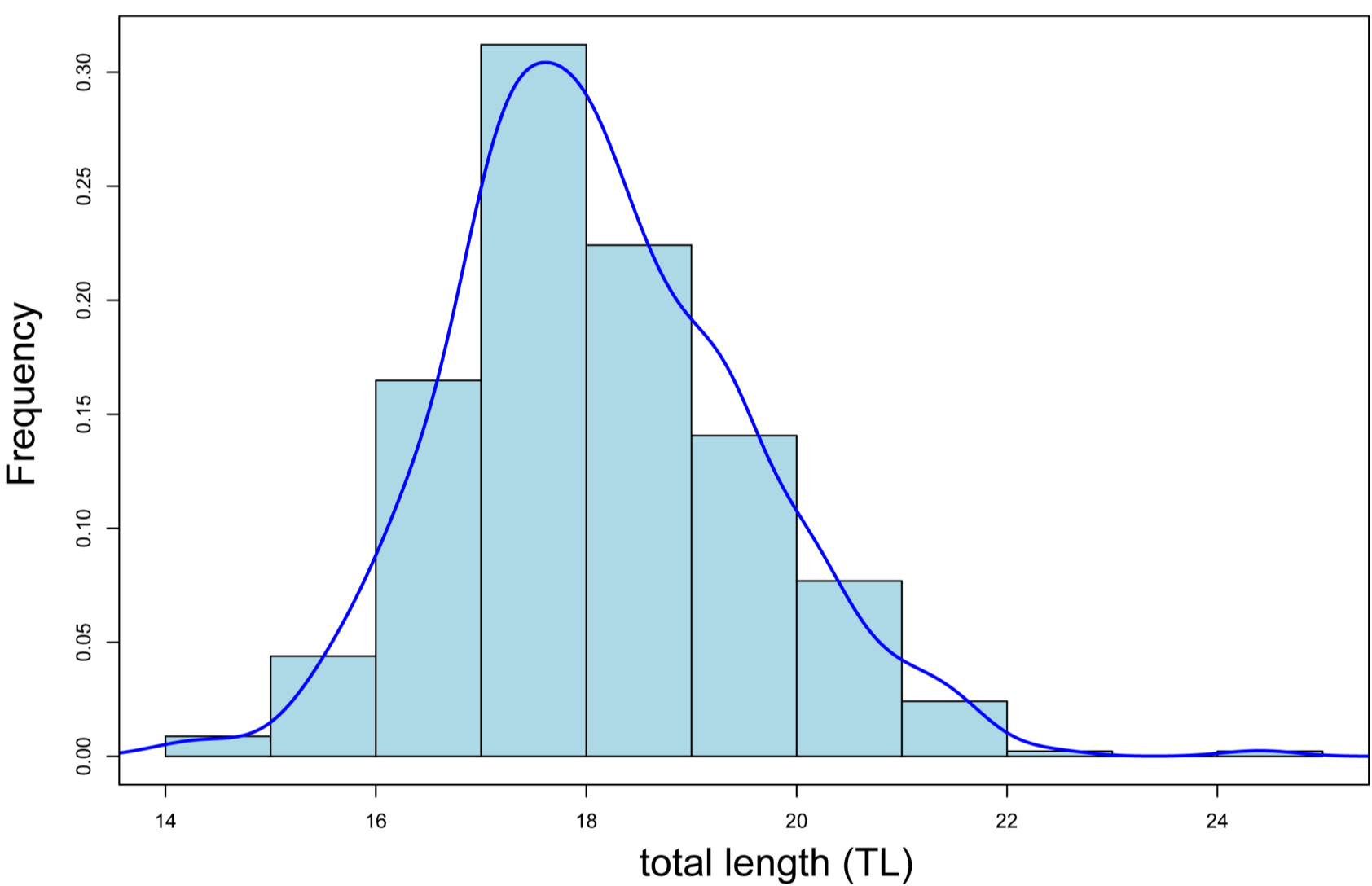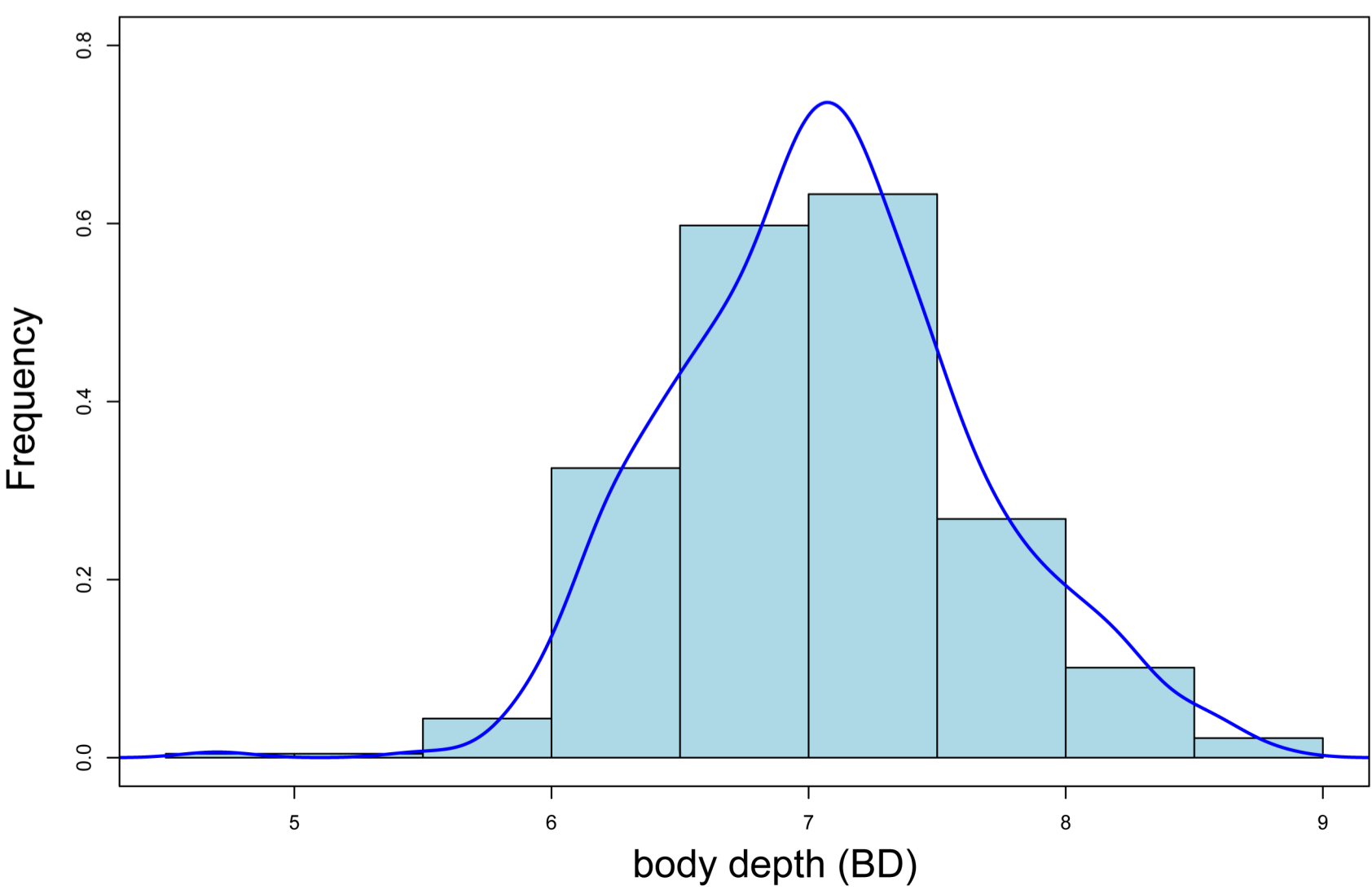

Supplement: Supplementary file 3 — Fig S3 [file EVA-15-523-s001.pdf]

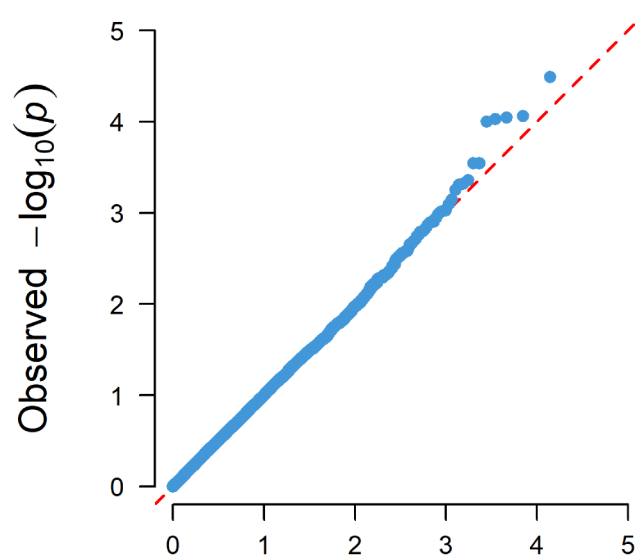

(a) Expected  $-\log_{10}(p)$

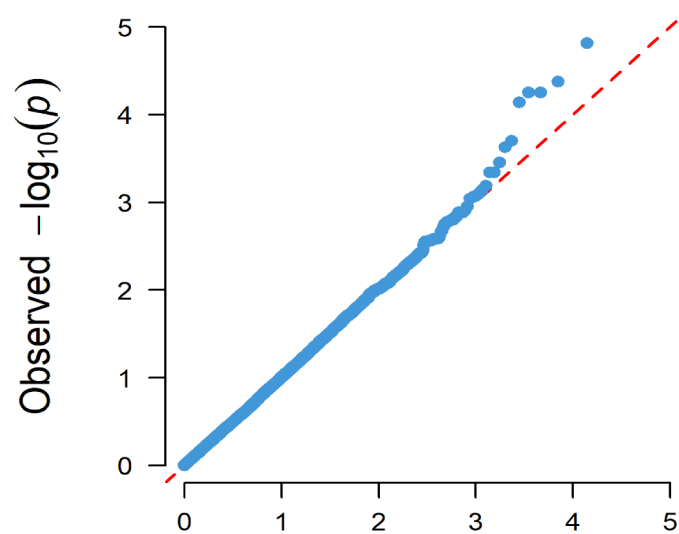

(b) Expected  $-\log_{10}(p)$

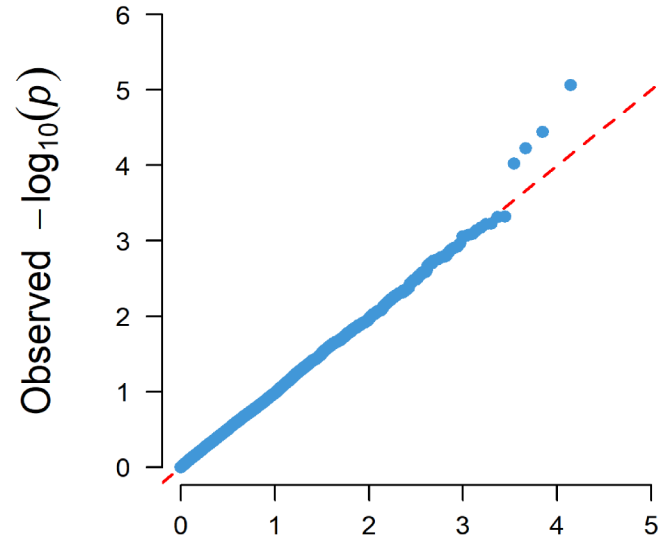

(c) Expected  $-\log_{10}(p)$

Supplement: Supplementary file 4 — Fig S4 [file EVA-15-523-s004.pdf]

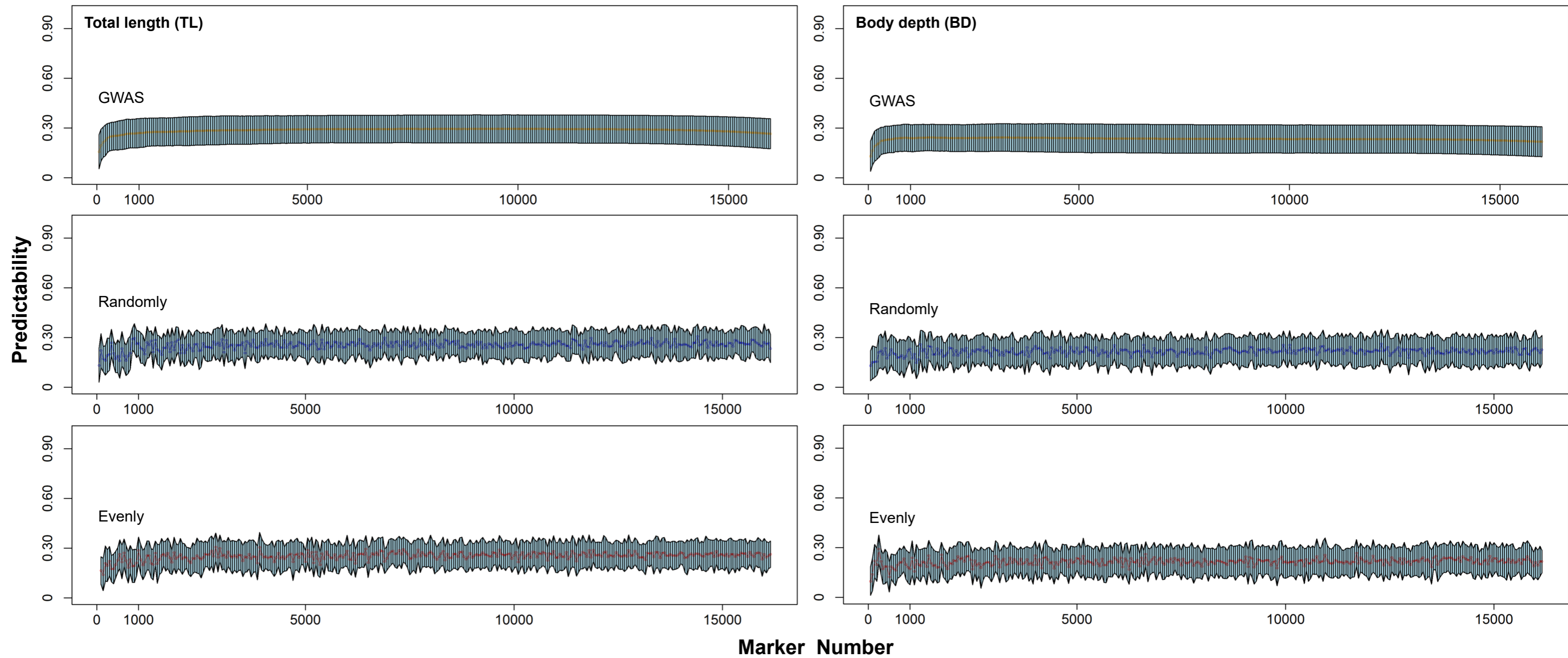

Supplement: Supplementary file 5 — Fig S5 [file EVA-15-523-s006.pdf]
