# Supplementary material for: First genomic prediction and genome‐wide association for complex growth‐related traits in Rock Bream (Oplegnathus fasciatus)
Source: Evol Appl. 2021 Mar 17;15(4):523–36. doi: 10.1111/eva.13218 (PMC9046763; doi:10.1111/eva.13218)
Supplement: Supplementary file 6 — Table S1 [file EVA-15-523-s002.docx]

Table S1. Genome-wide suggestive SNPs associated with growth traits identified by GWAS analysis.

| **Trait** | **SNP** | **CHR** | **Position (bp)** | ***P*-value** | **Major_allele** | **Minor_allele** | **Beta (*P*)** | **SE (Beta *P*)** | **PVE (%)** |
| --- | --- | --- | --- | --- | --- | --- | --- | --- | --- |
| **BW** | SNP12834 | 3 | 20141071 | 3.26E-05 | T | C | 1.66E+01 | 4.74E+00 | 7.55 |
|  | SNP11178 | 13 | 15312027 | 8.73E-05 | C | G | 1.52E+01 | 4.13E+00 | 7.14 |
|  | SNP33570 | 14 | 2783452 | 9.01E-05 | G | T | -1.41E+01 | 3.82E+00 | 7.13 |
|  | SNP30417 | 16 | 17024879 | 9.41E-05 | C | T | -9.75E+00 | 2.82E+00 | 7.11 |
|  | SNP12835 | 3 | 20151776 | 9.98E-05 | T | C | 1.58E+01 | 4.76E+00 | 7.09 |
| **TL** | SNP27877 | 20 | 3385112 | 1.54E-05 | T | C | 4.47E-01 | 1.31E-01 | 6.45 |
|  | SNP12834 | 3 | 20141071 | 4.19E-05 | T | C | 7.28E-01 | 2.03E-01 | 6.03 |
|  | SNP26915 | 24 | 22497710 | 5.58E-05 | A | G | 5.51E-01 | 1.51E-01 | 5.83 |
|  | SNP28762 | 24 | 22820802 | 5.58E-05 | A | T | -4.27E-01 | 1.46E-01 | 5.83 |
|  | SNP15114 | 19 | 13032228 | 7.25E-05 | A | G | 6.83E-01 | 2.21E-01 | 5.69 |
| **BD** | SNP29166 | 14 | 6503024 | 8.72E-06 | T | G | 3.24E-01 | 8.39E-02 | 7.27 |
|  | SNP17210 | 16 | 13498230 | 3.62E-05 | A | G | 3.37E-01 | 8.66E-02 | 6.66 |
|  | SNP26114 | 16 | 1519759 | 6.07E-05 | G | A | 3.10E-01 | 8.80E-02 | 6.44 |
|  | SNP27877 | 20 | 3385112 | 9.57E-05 | T | C | 1.86E-01 | 5.13E-02 | 6.25 |

PVE: phenotypic variances explained.
