# Supplementary material for: First genomic prediction and genome‐wide association for complex growth‐related traits in Rock Bream (Oplegnathus fasciatus)
Source: Evol Appl. 2021 Mar 17;15(4):523–36. doi: 10.1111/eva.13218 (PMC9046763; doi:10.1111/eva.13218)
Supplement: Supplementary file 7 — Table S2 [file EVA-15-523-s007.docx]

Table S2. Summary of growth-related candidate genes identified from GWAS in *O. fasciatus*.

| **Trait** | **SNP** | **CHR** | **Gene-symbol** | **Gene Annotation** |
| --- | --- | --- | --- | --- |
| **BW** | SNP12834 | 3 | NRAC | Nutritionally-regulated adipose and cardiac-enriched protein homolog |
|  | SNP11178 | 13 | DPP10 | Inactive dipeptidyl peptidase 10 |
|  |  |  | SL9A2 | Sodium/hydrogen exchanger 2 (H7) |
|  | SNP33570 | 14 | HACE1 | E3 ubiquitin-protein ligase HACE1 |
|  | SNP30417 | 16 | RNF152 | E3 ubiquitin-protein ligase RNF152 |
|  | SNP12835 | 3 | KIF26A | Kinesin-like protein KIF26A |
| **TL** | SNP27877 | 20 | HUTH | Histidine ammonia-lyase |
|  |  |  | ELK3 | ETS domain-containing protein Elk-3 |
|  |  |  | CDK17 | Cyclin-dependent kinase 17 |
|  |  |  | UCMA | Unique cartilage matrix-associated protein |
|  |  |  | Camk1d | Calcium/calmodulin-dependent protein kinase type 1D |
|  |  |  | DHTKD1 | Probable 2-oxoglutarate dehydrogenase E1 component DHKTD1 |
|  |  |  | CELF2 | CUGBP Elav-like family member 2 |
|  | SNP12834 | 3 | NRAC | Nutritionally-regulated adipose and cardiac-enriched protein homolog |
|  | SNP26915 | 24 | HGFA | Hepatocyte growth factor activator |
|  |  |  | Rgs12 | Regulator of G-protein signaling 12 |
|  |  |  | MSANTD1 | Myb/SANT-like DNA-binding domain-containing protein 1 |
|  |  |  | DTX4 | E3 ubiquitin-protein ligase DTX4 |
|  |  |  | OSH1 | Oxysterol-binding protein homolog 1 |
|  | SNP28762 | 24 | CAPN1 | Calpain-1 catalytic subunit |
|  | SNP15114 | 19 | CNTNAP5 | Contactin-associated protein-like 5 |
| **BD** | SNP29166 | 14 | SYBU | Syntabulin |
|  |  |  | RCAS1 | Receptor-binding cancer antigen expressed on SiSo cells |
|  |  |  | TTK | Dual specificity protein kinase TTK |
|  |  |  | ODBB | 2-oxoisovalerate dehydrogenase subunit beta, mitochondrial |
|  | SNP17210 | 16 | CARD8 | Caspase recruitment domain-containing protein 8 |
|  |  |  | CASP1 | Caspase-1 |
|  | SNP26114 | 16 | CUBN | Cubilin |
|  |  |  | ZCCHC8 | Zinc finger CCHC domain-containing protein 8 |
|  | SNP27877 | 20 | UCMA | Unique cartilage matrix-associated protein |
|  |  |  | Camk1d | calcium/calmodulin-dependent protein kinase type 1D-like |
|  |  |  | DHTKD1 | Probable 2-oxoglutarate dehydrogenase E1 component DHKTD1 |
